# Long lasting MDM2/Translocator protein modulator: a new strategy for irreversible apoptosis of human glioblastoma cells

**DOI:** 10.18632/oncotarget.6872

**Published:** 2016-01-09

**Authors:** Simona Daniele, Elisabetta Barresi, Elisa Zappelli, Luciana Marinelli, Ettore Novellino, Federico Da Settimo, Sabrina Taliani, Maria L. Trincavelli, Claudia Martini

**Affiliations:** ^1^ Department of Pharmacy, University of Pisa, Pisa, Italy; ^2^ Department of Pharmacological and Biomolecular Sciences, University of Milan, Milan, Italy; ^3^ Department of Pharmacy, University of Naples Federico II, Napoli, Italy

**Keywords:** murine double minute 2, translocator protein, long-lasting inhibitors, glioblastoma multiforme, extracellular signal regulated kinases

## Abstract

The development of multi-target drugs and irreversible modulators of deregulated signalling proteins is the major challenge for improving glioblastoma multiforme (GBM) treatment. Reversible single-target drugs are not sufficient to sustain a therapeutic effect over time and may favour the activation of alternative signalling pathways and the onset of resistance phenomena. Thus, a multi-target compound that has a long-lasting mechanism of action might have a greater and longer life span of anti-proliferative activity. Recently, a dual-target indol-3ylglyoxyldipeptide derivative, designed to bind to the Translocator Protein (TSPO) and reactivate p53 function via dissociation from its physiological inhibitor, murine double minute 2 (MDM2), has been developed as a potent GBM pro-apoptotic agent. In this study, this derivative was chemically modified to irreversibly bind MDM2 and TSPO. The new compound elicited a TSPO-mediated mitochondrial membrane dissipation and restored p53 activity, triggering a long-lasting apoptosis of GBM cells. These effects were sustained over time, involved a stable activation of extracellular signal regulated kinases and were specifically observed in cancer cells, in which these protein kinases are deregulated. Dual-targeting and irreversible binding properties combined in the same molecule may represent a useful strategy to overcome the time-limited effects elicited by classical chemotherapies.

## INTRODUCTION

Glioblastoma multiforme (GBM), a WHO grade IV malignant glioma [1], is the most common and lethal primary brain tumour in adults [2], with a median survival of less than 2 years. Presently, treatment of GBM is based on cytoreductive resection combined with radiotherapy and adjuvant chemotherapy with Temozolomide (TMZ) [3]. Nonetheless, the prognosis of GBM patients remains poor because of the onset of drug resistance and tumour recurrence [4, 5]. A number of attempts have been made to understand the molecular basis of resistance, and consequently, different approaches have been tried to overcome this phenomenon such as the use of multi-target drugs and the trial of long-lasting active molecules [6]. Recent discoveries in molecular biology have shown that the aberration of different signalling pathways are involved in the pathogenesis of malignant gliomas and have allowed the identification of new targets for novel therapeutic approaches, including growth factor ligands, receptors, and intracellular downstream effectors [7]. Because these deregulated intracellular signalling pathways, including the PI3K/Akt/mammalian target of the rapamycin (mTOR) and the Ras/extracellular signal-regulated kinase (ERK), are a point of convergence for different stimuli, the concept of one disease-one drug is challenging, and multi-target therapy is becoming the favourite way to develop innovative and more efficient therapies [8-11].

Furthermore, it has been postulated that reversible drugs may have several limitations in cancer therapy. Indeed, a reversible molecule may not be enough to sustain a therapeutic effect over time, thereby favouring the activation of alternative signalling pathways that escape drug action and cause resistance. For these reasons, oncology research has recently been focused on the synthesis and development of new irreversible and long-lasting molecules [12]. Recently, different irreversible inhibitors for tyrosine kinase receptors have been described, and some of these are under clinical development for the therapy of breast, lung and other solid tumours [13-18]. Lately, pre-clinical results for an irreversible, orally administered pan-ERBB inhibitor in mice GBM xenograft tumours have been reported [19]. These data showed that the long-lasting activity of the drug towards epidermal growth factor receptor (EGFR) can substantially increase the efficacy of the drug, suggesting irreversible ligands as a new, valuable therapeutic option to overcome drug resistance.

In GBM, p53 and Translocator protein (TSPO), both acting as apoptosis inducers, represent two attractive intracellular targets. Classic TSPO ligands, such as PK11195, have demonstrated anticancer effects both *in vit*ro and *in vivo*; in addition, newly synthesized TSPO ligands triggered apoptosis in human and rat GBM cells by modulating the opening of the mitochondrial permeability transition pore (MPTP), of which TSPO is an important constitutive protein [20, 21].

On the other hand, deregulation of the p53 protein is widely described in the literature, and reactivation of its endogenous function represents an important anticancer therapeutic strategy, at least for those tumours that do not contain a mutant p53. P53 is negatively regulated by the murine double minute 2 (MDM2), and inhibitors of MDM2/p53 interaction currently represent another viable approach in GBM therapy [22-24].

Recently, we have characterized TSPO/MDM2 dual-target ligands and demonstrated that these agents present an attractive multi-modal anti-cancer activity in GBM cells [25]. In this study, we started from this scaffold and synthesized a new dual target compound with long-lasting binding properties towards MDM2 and TSPO. Specifically, this compound was characterized by the presence of a chemo-reactive isothiocyanate group, able to covalently bind the target proteins. The compound activated mitochondrial membrane dissipation and restored p53 activity, inducing GBM cell apoptosis. It is noteworthy that the new molecule oriented the cellular fate towards irreversible apoptosis via the stable activation of the p53-MDM2-ERK axis. These results indicated that the development of new molecules targeting more key proteins and able to regulate intracellular signalling over time may offer innovative and alternative strategies to overcome the reversible activity of chemotherapy agents.

## RESULTS

### Synthesis of the new compound

**Figure 1:**
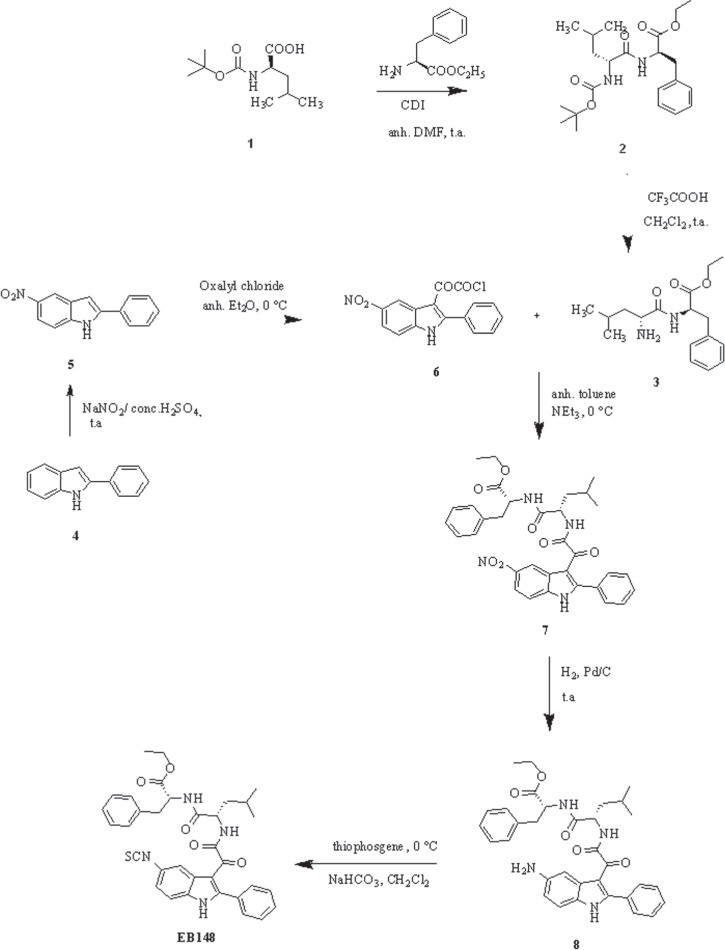
Synthesis of the new derivative EB148

The convergent procedure applied for the synthesis of the target indole derivative EB148 is outlined in Figure 1. The dipeptide 3 was obtained in two steps starting from Boc-L-leucine 1, which was condensed with L-phenylalanine ethyl ester in the presence of *N,N*’-carbonyldiimidazole (CDI) in anhydrous dimethylformamide to obtain compound 2, then deprotected by treatment with trifluoroacetic acid, as previously described [25]. Acylation of the 5-nitro-2-phenylindole 5 with oxalyl chloride in anhydrous ethyl ether at 0°C yielded the corresponding 5–nitro-2-phenylindolylglyoxylyl chloride 6 [26].

Reaction of derivative 6 with compound 3 in the presence of triethylamine in anhydrous toluene at 0°C gave compound 7, which was purified by flash chromatography. Then, the (5-nitro-2-phenylindol-3-yl)glyoxyl-L-leucine-L-phenylalanine ethyl ester 7 was catalytically hydrogenated over palladium to yield the corresponding amine 8, which was then reacted with thiophosgene in a biphasic CH_2_Cl_2_/aqueous NaHCO_3_ solution to give the desired isothiocyanato derivative EB148, which was purified by flash chromatography. The reversible analogue EB54 was synthesized essentially as described previously [25]. Purity degree of the compounds (> 96%) was assessed by HPLC.

The stability of EB148 in aqueous solution was evaluated by HPLC analysis. The results showed no significant degradation of the compound: indeed, the average peak area of EB148 was 98.83 ± 0.47 % within 72 h.

### EB148 exhibited long-lasting binding properties to TSPO

**Figure 2:**
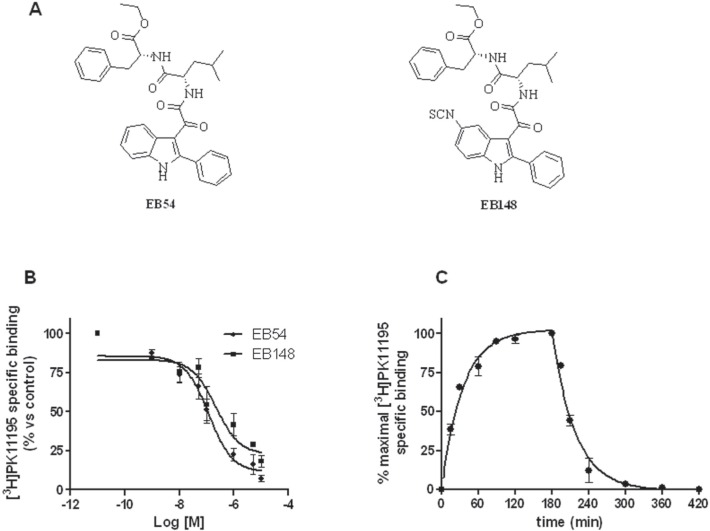
Affinity to TSPO in [**^3^**H]PK11195 radioligand binding assay **A**. Chemical structures of EB54 and EB148. **B**. U87MG cell membranes (20 μg of proteins) were incubated with 1.5 nM [^3^H]PK11195 and different compound concentrations. After reaching equilibrium, samples were filtered and bound radioactivity was counted. Data are expressed as a percentage of specific binding versus the basal value (set to 100%) and represent the mean ± SEM of three different experiments. **C**. Kinetics binding profile of [^3^H]PK11195 to TSPO. U87MG cell membranes (20 μg protein) were incubated with 1.5 nM radioligand at various times up to 3 h. Dissociation kinetics were determined by pre-equilibrating membranes and [^3^H]PK11195 for 180 min; a saturating concentration of cold PK11195 (1 μM) was then added at various times. Association and dissociation data are expressed as percentage of maximum specific binding, and globally fit using Prism 5.0. Data represent the mean ± SEM of three different experiments.

At first, the ability of EB148 to bind human TSPO was tested by radioligand binding studies in U87MG cells. EB54 and EB148 (Figure 2A) displaced specific [^3^H]PK11195 binding in a concentration-dependent manner, with Ki values of 108 ± 10 nM and 199 ± 18 nM (Figure 2B), respectively. The affinity of the reversible ligand (EB54) towards human TSPO was consistent with data obtained in rat kidney membranes [25].

Direct kinetic binding studies indicated that [^3^H]PK11195 labelled TSPO with an association constant rate (Kon) of 9.15 ± 0.85 × 10^6^ M^−1^ min^−1^ (Figure 2C; Table 1). Dissociation occurred rapidly when the ligand-receptor complex was exposed to a saturating concentration of unlabelled PK11195 (Figure 2C). The dissociation rate constant (Koff) was estimated to be 0.0264 ± 0.0018 min^−1^, leading to a calculated half-life of 26.1 ± 1.8 min^−1^.

**Table 1:** Kinetic binding parameters of various compounds at human TSPO

Compound	Kon (M-1 min-1)	Koff (min-1)	t1/2	pKd	pKi (nM)
PK11195^a^	9.15 ± 0.85 × 10^6^	0.0264 ± 0.0018	26.1 ± 1.8	2.89 × 10^−9^	2.88 ± 0.13
EB54	2.16 ± 0.31 × 10^5^	0.0289 ± 0.0031	23.9 ± 2.6	134 × 10^−9^	108 ± 10
EB148	3.21 ± 0.35 × 10^5^	0.004809 ± 0.00059	144 ± 5	150 × 10^−9^	199 ± 18

a All the kinetic data were obtained from competition kinetic studies, with the exception of [^3^H] PK11195 that was obtained through direct association and dissociation measurements (Figure 1). Data represent the mean ± SEM of three different experiments.

**Figure 3:**
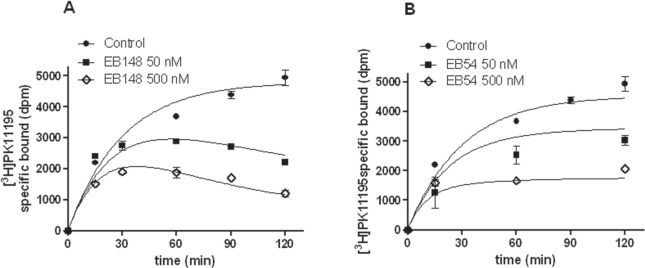
Competition kinetics curves for [**^3^**H]PK11195 binding to TSPO-expressing membranes U87MG cell membranes (20 μg protein) were incubated with 1.5 nM radioligand at various times in the presence of increasing concentrations of EB54 or EB148. Data are expressed as [^3^H]PK11195 specific bound (dpm) and are representative of at least three independent experiments, each performed in duplicate.

One of the advantages of long-lasting inhibitors is the sustained inhibition of the target protein, with a recovery of activity that occurs only after re-synthesis of the target protein. To determine the binding kinetics of EB54 and EB148, a competition kinetic method was performed [27,28]. Figure 3 shows the effect of multiple concentrations of unlabelled test compounds on the association binding kinetics of [^3^H]PK11195. By fixing the association and dissociation rate constants for [^3^H]PK11195 to the values previously determined, it was possible to obtain the relevant kinetic binding parameters for each compound (Table 1).

The rank order of dissociation half-lives for the tested compounds was EB148 > PK11195 ≥ EB54, thus demonstrating that EB148 acts as a long-lasting TSPO ligand. To verify the kinetic approach, the pKd values for the tested ligands were calculated as a ratio of estimated koff and kon values. The comparison between pKd and competition pKi values (Table 1 and Figure 2B) demonstrates that the two methods produce comparable results, thus validating the indirect kinetic method.

### EB148 induced a sustained membrane potential dissipation

**Figure 4:**
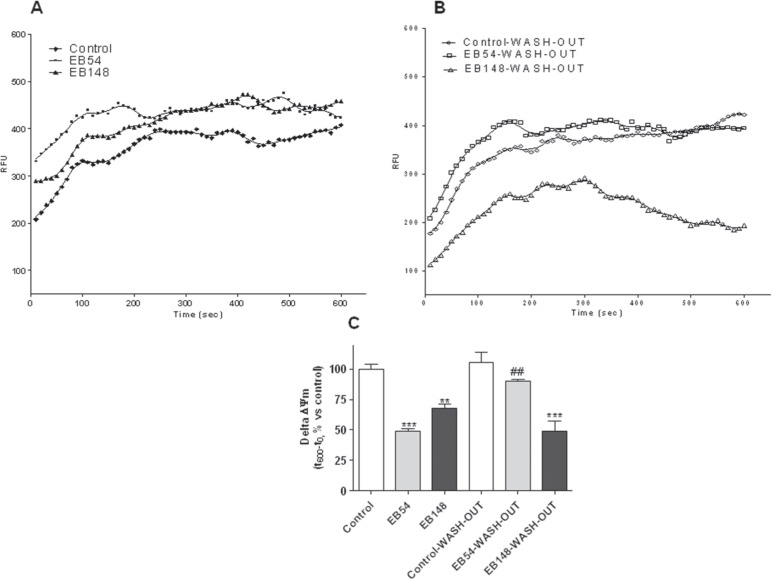
Evaluation of Δψm in isolated mitochondria Mitochondria (5 μg of proteins) were treated with DMSO, EB54 or EB148 for 20 min. In wash-out experiments, mitochondria were then centrifuged at 11.000 × g for 5 min, washed with fresh saline and suspended in JC-1 buffer not containing the drugs. The Δψm was evaluated using the JC-1 protocol as described in the Methods section. **A**., **B**. Representative graph of mitochondria potential evaluation using the JC-1 protocol. The results were expressed as RFU units in time. **C**. The data are expressed as variation of JC1 uptake into mitochondria, calculated as the difference between the RFU read after 10 minutes and the RFU read at the beginning, and represent the mean value ± SEM of three different experiments. Statistical significance was determined with a one-way ANOVA with a Bonferroni post-test: **p<0.01, ***p<0.001 vs. control; ##p<0.01 vs. EB54-treated cells.

Several pieces of evidence have shown that TSPO is a constituent of the MPTP and therefore takes part in the dissipation of membrane potential (Δψm) that occurs after MPTP opening. To assess whether the compounds could modulate MPT-pore opening through their selective binding to TSPO, Δψm was measured in mitochondria isolated from U87MG cells. The treatment of the isolated mitochondria with EB54 or EB148 (1 μM) for 20 min led to a significant reduction in tetrachloro-tetraethylbenzimidazolylcarbocyanine iodide (JC-1) accumulation compared to control untreated mitochondria, demonstrating the collapse of Δψm (Figure 4A, 4B and 4C). When compound-treated mitochondria were centrifuged and washed to remove the TSPO ligands, only EB148 determined Δψm collapse. These results demonstrate that the long-lasting TSPO ligand can dissipate Δψm even after mitochondria wash-out (Figure 4B and 4C).

### EB148 induced a long-lasting dissociation of p53-MDM2 complex

**Figure 5:**
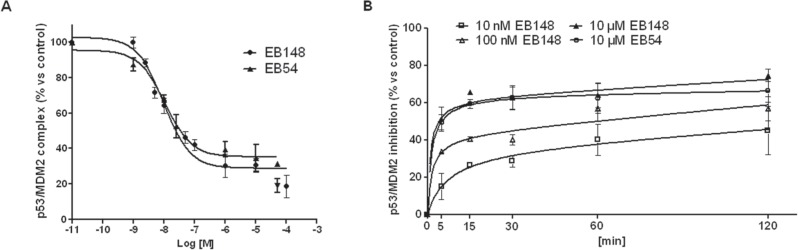
Affinity and association kinetics of EB148 towards MDM2 **A**. U87MG cell lysates containing the native MDM2/p53 complex were pre-incubated with DMSO (control) or different concentrations of EB54 or EB148 for 15 min. Then the lysates were captured on wells pre-coated with MDM2 antibody. After extensive washing, the levels of the MDM2/p53 complex were quantified using an antibody specific for p53 and subsequently an HRP-conjugated antibody and a TMB substrate kit. Blank wells were obtained in the absence of p53 antibody. Data are expressed as a percentage of control set to 100%, and represent the mean ± SEM of three independent experiments. **B**. U87MG cell lysates containing the native MDM2/p53 complex were pre-incubated with DMSO (control) or the indicated concentrations of EB148 or EB54 for different times (0-120 min). Following incubation, the lysates were captured on wells pre-coated with MDM2 antibody and levels of the MDM2/p53 complex were quantified as in A. Curves were generated using a sigmoidal dose-response curve model (A) (GraphPad Prism 5 software) from which the IC_50_ values were derived, or an association kinetics model (B).

The activity of EB148 in inhibiting MDM2-p53 complex was assessed using a validated assay [25, 29]. The compound was able to efficaciously dissociate the MDM2-p53 complex with an IC_50_ of 6.81 ± 0.79 nM (Figure 5A), a value comparable to that obtained with the reversible analogue EB54 [25]. The effect of the compound, tested at three different concentrations, appeared to be time and concentration dependent, with a half-life (t_1/2_) of 16.4 ± 2.1 (10 nM), 3.83 ± 0.65 (100 nM) and 2.27 ± 0.93 (10 μM) min^−1^, respectively. No significant differences in the association kinetics between EB54 and EB148, both tested at a 10 μM concentration, were detected (EB54: t_1/2_=2.33 ± 0.83; Figure 5B).

**Figure 6:**
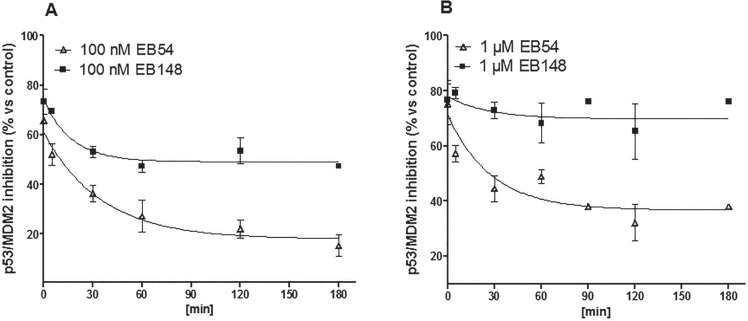
Dissociation Kinetics from MDM2 (**A**., **B**.) Cell lysates containing the native MDM2/p53 complex were pre-incubated with DMSO (control) or the indicated concentrations of EB148 or EB54 for 120 min, to reach an association plateau. Dissociation was initiated by the addition of 20 volumes of cold buffer and the lysates were further incubated at 4°C for the indicated times (0-180 min). Following incubation, 200 μl of the mixture were captured on wells pre-coated with MDM2 antibody. After extensive washing, the levels of the MDM2/p53 complex were quantified using an antibody specific for p53 and subsequently an HRP-conjugated antibody and a TMB substrate kit. Data are expressed as a percentage of control set to 100% and represent the mean ± SEM of three independent experiments. Curves were generated using a dissociation kinetics model (GraphPad Prism 5 software).

To verify the EB148 covalent mechanism of action, kinetic dissociation of the p53/MDM2 complex was evaluated in cell lysates and in whole cells. Cell lysates were treated for 60 min with EB148 or EB54 at a concentration ten-fold higher than their IC_50_ value. Afterwards, the lysates were diluted 1:20 by adding reaction buffer and incubated for different times (0-180 min), after which the extent of p53-MDM2 complex was quantified. The activity of compound EB148 in inhibiting p53-MDM2 complex was maintained unchanged after sample dilution. In contrast, the reversible analogue gradually decreased its activity through the dilution time (Figure 6A and 6B). These data confirmed that EB148 acts as a long-lasting inhibitor of the p53-MDM2 complex.

**Figure 7:**
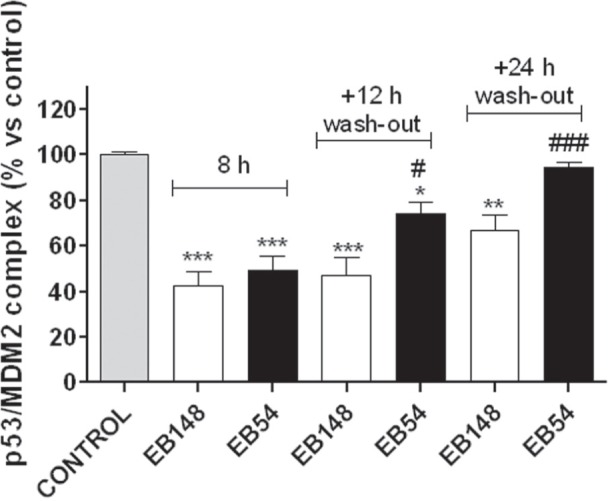
Levels of p53/MDM2 complex in U87MG whole cells U87MG cells were treated with DMSO (control), 1 μΜ EB148 or 1 μΜ EB54 for 8 h; when indicated, cells were washed-out for 12-24 h to remove the drugs. At the end of the treatments, the cells were collected and lysed; the quantification of p53/MDM2 complexes in U87MG cells was assessed by the ELISA method as described above. Data are expressed as percentage of control set to 100%, and represent the mean ± SEM of three independent experiments. The significance of the differences was determined with a one-way ANOVA with a Bonferroni post-test: * p<0.05, ** p<0.01, *** p<0.001 vs. control; # p<0.05, ### p<0.001 vs. cells treated for 8 h.

A limitation of the “in tube” assay is that it is an isolated system that does not account for compound permeability through the cell membrane as well as the cellular machinery that may interfere with the biological activity by regulating the expression/degradation of protein target and of the compound itself. For this reason, the activity of EB148 or EB54 as inhibitors of the p53-MDM2 complex was also assessed in whole cells using the same validated ELISA assay. U87MG cells were incubated for 8 h with EB148 or EB54 and then the levels of the p53-MDM2 complex were quantified. As expected, both EB54 and EB148 inhibited the p53-MDM2 complex after 8 h (Figure 7). When U87MG cells were washed out to remove ligands, a significant difference in the recovery of p53-MDM2 complex formation was observed. As shown in Figure 7, in cells treated with EB54, the recovery of p53-MDM2 complex occurred within 12 h. The long-lasting compound EB148 significantly slowed down new complex formation, which was maintained significantly lower than in the control even after 24 h cell wash-out (Figure 7).

### EB148 efficiently reactivated the p53 pathway

**Figure 8:**
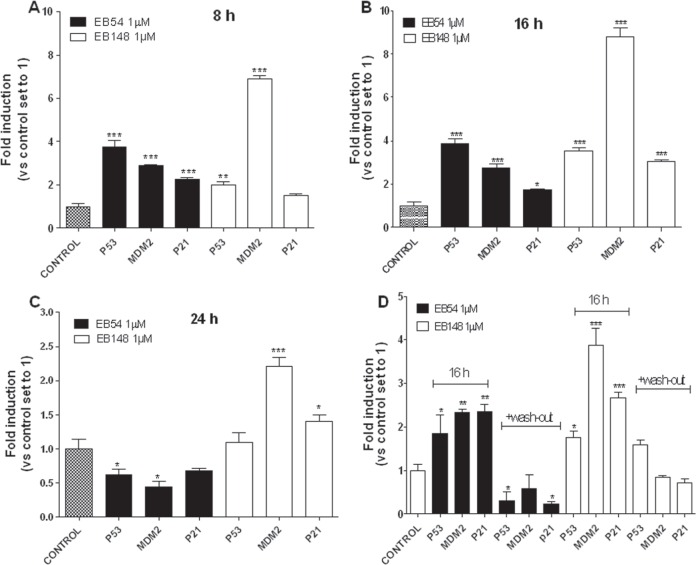
Effects of the MDM2 inhibitors on the reactivation of p53 pathway U87MG cells were treated with DMSO (control), or 1 μΜ EB148 or 1 μΜ EB54 for 8 h **A**., 16 h **B**., or 24 h **C**. Aliquots of cells, after treatment with the compounds for 16 h, were washed-out for additional 16 h in drug-free medium **D**. At the end of the treatment period, the relative mRNA quantification of p53 target genes (p53, p21 and MDM2) was performed by real-time RT-PCR as described in the Methods section. The data are the mean values ± SEM of three different experiments, each performed in duplicate. The significance of the differences was determined with a one-way ANOVA with a Bonferroni post-test: * p<0.05, ** p<0.01, *** p<0.001 vs. control.

To support the reactivation of the p53 pathway, mRNA expression levels of p53 target genes and p53 protein expression were evaluated. Significant differences in the kinetics of induction of p53 target genes were detected after cell incubation with the long-lasting MDM2 inhibitor EB148 or the reversible analogue EB54. Indeed, after 8 and 16 h of U87MG cell treatment EB54 induced a significant increase in the mRNA expression level of p21, p53 and MDM2 (Figure 8A and 8B). The expression of these genes gradually slowed down and, after 24 h, reached values significantly lower than controls (Figure 8C), likely by means of a feed-back inhibition mechanism.

In contrast, EB148 induced a sustained increase in the mRNA expression of p53 target genes up to 24 h of cell treatment. Notably, high induction of MDM2 mRNA was observed: EB148 caused a 6.9-, 8.8- and 2.2-fold induction of MDM2 mRNA after 8, 16 and 24 h of cell treatment, respectively, whereas the maximal increase of the MDM2 transcript elicited by EB54 was 2.9-fold versus control (Figure 8A, 8B and 8C). To evaluate reversible versus long-lasting effects of EB54 and EB148, cells were treated with each compound for 2 h and then washed out for 16 h in a drug-free medium. After the cells were challenged with EB54, the levels of the p53 target genes were down-regulated (Figure 8D). When the long-lasting compound EB148 was added in the pre-incubation medium, the transcripts of p53 target genes returned to control values (Figure 8D).

**Figure 9:**
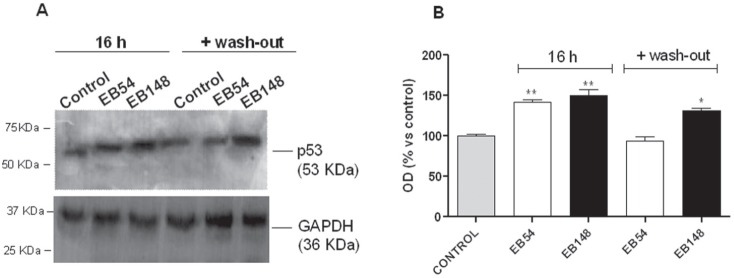
Effects of the MDM2 inhibitors on p53 protein accumulation U87MG cells were treated with DMSO (control), or 1 μM EB148 or 1 μΜ EB54 for 16 h, followed by a wash-out period of an additional 16 h. At the end of the treatment, p53 protein levels were evaluated by western blotting analysis using GAPDH as the loading control. **A**. Representative western blots. **B**. Densitometric analysis of the immunoreactive bands performed using the ImageJ program. The data were expressed as the percentage relative to control, and they are the mean values ± SEM of three different experiments. The significance of the differences was evaluated using a one-way ANOVA with the Bonferroni post-test: *p<0.05, **p<0.01 vs. control.

Consistent with the data obtained with Real time PCR analysis (RT-PCR), challenging GBM cells with EB54 and EB148 for 16 h led to a slight but significant increase in p53 protein levels (Figure 9A and 9B); a wash-out period of 16 h brought p53 accumulation to control levels in EB54-treated cells. In contrast, even if the transcription was not activated at this time point, EB148 also maintained high p53 levels after cell wash-out (Figure 9A and 9B).

### EB148 induced a long-lasting activation of intracellular ERK

**Figure 10:**
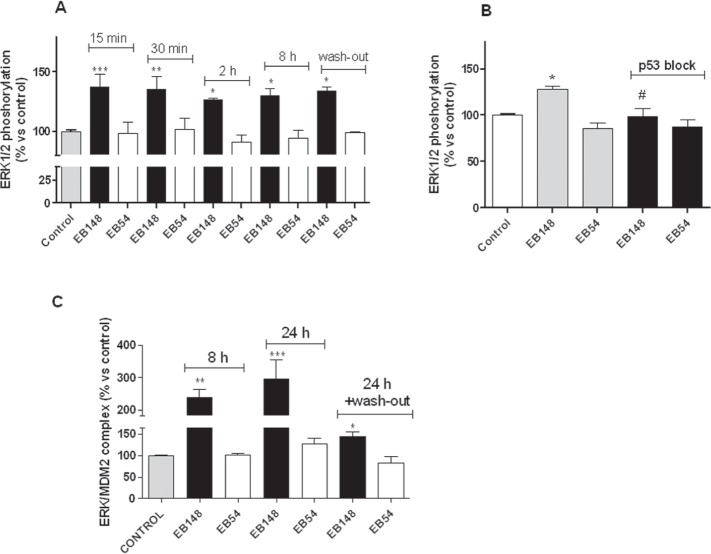
ERK phosphorylation and levels of ERK/MDM2 complex in U87MG cells **A**. U87MG cells were treated with DMSO (control), 1 μΜ EB148 or 1 μΜ EB54 for the indicated times; in some experiments, after 2 h of treatment the medium was replaced by fresh medium not containing drugs for an additional 2 h. At the end of the treatment period the levels of ERK 1/2 phosphorylation were evaluated using ELISA kits, as described in the Methods section. The data are expressed as the percentage of phosphorylated ERK1/2 versus untreated cells (control), which were set at 100%. Data are the mean values ± SEM of three independent experiments performed in triplicate. Analysis of significant differences in mean ERK/MDM2 complex levels was performed using one-way ANOVA with a Bonferroni post-test: * p< 0.05, ** p< 0.01, *** p<0.001 vs. control. **B**. U87MG cells were treated for 30 min with DMSO (control) or the p53 inhibitor, pifithrin-β (1 μΜ); subsequently, cells were incubated with DMSO, EB148 or EB54 1 μM for 2 h. At the end of the treatment periods, the levels of ERK 1/2 phosphorylation were evaluated using ELISA kit, as in A. The significance of differences was performed using one-way ANOVA with a Bonferroni post-test: * p< 0.05 vs. control; #p<0,05 vs. cells not treated with the p53 inhibitor. **C**. U87MG cells were treated with DMSO (control), 1 μΜ EB148 or 1 μΜ EB54 for the indicated times; in some experiments, after 8 h of treatment, the medium was replaced by fresh medium not containing drugs for an additional 24 h. At the end of the treatment period, U87MG cells were collected and suspended in lysis buffer. Equal amounts of cell lysates were captured on wells pre-coated with MDM2 antibody. After extensive washing, the levels of the ERK/MDM2 complex were quantified using an antibody specific for ERKs, and subsequently an HRP-conjugated antibody and a TMB substrate kit. Data are expressed as percentage of control set to 100%, and represent the mean ± SEM of three independent experiments. The significance of the differences was determined with a one-way ANOVA with Bonferroni post-test: * p<0.05, ** p<0.01, *** p<0.001 vs. control.

The kinetic pattern of ERK activation is the pre-requisite that regulates the final biological outcomes of these kinases. A transient kinase activation is commonly associated with a proliferative effect, whereas their sustained phosphorylation over time triggers death signalling pathways. In this regard, the kinetics of ERK activation induced by EB148 and its reversible analogue, EB54, were evaluated. The data depicted in Figure 10A and 10B showed that reversible compound EB54 did not induce any significant ERK1/2 stimulation. In contrast, EB148 induced significant ERK phosphorylation, and this signal remained switched-on up to 8 h after cell treatment (Figure 10A). Moreover, after cell wash-out the long lasting compound EB148 continued to significantly activate the MAPKs (Figure 10A).

To unequivocally prove that ERK activation elicited by EB148 was mediated by p53, experiments were repeated in the presence of the p53 inhibitor pifithrin-β (1 μM) [30].

As depicted in Figure 10B, blocking p53 completely impaired EB148-mediated ERK1/2 activation but did not significantly affect the EB54 response. These data demonstrate that the sustained ERK1/2 phosphorylation elicited by the long-lasting MDM2 inhibitor involves a p53-mediated mechanism.

It has been ascertained that a sustained activated ERK directly interacts with MDM2 inhibiting its ubiquitin ligase activity and triggering pro-apoptotic p53-mediated pathways [31,32]. To evaluate the role of ERK in the long-lasting effects evoked by EB148, the association between ERKs and MDM2 in response to cell treatment with EB148 was evaluated. EB148 induced a significant and marked induction of complex formation (Figure 10C). This association was evident after both 8 and 24 h after cell treatment and remained significant after cell wash-out for 24 h. These data suggest that the long-lasting inhibition of MDM2 induced sustained ERK phosphorylation, and this activated kinase might play a role in controlling MDM2 activity and triggering p53-mediated apoptotic effects. In contrast, cell incubation with EB54 did not induce any significant MDM2-ERK association.

### EB148 inhibited GBM cell proliferation over time and triggered apoptosis

**Figure 11:**
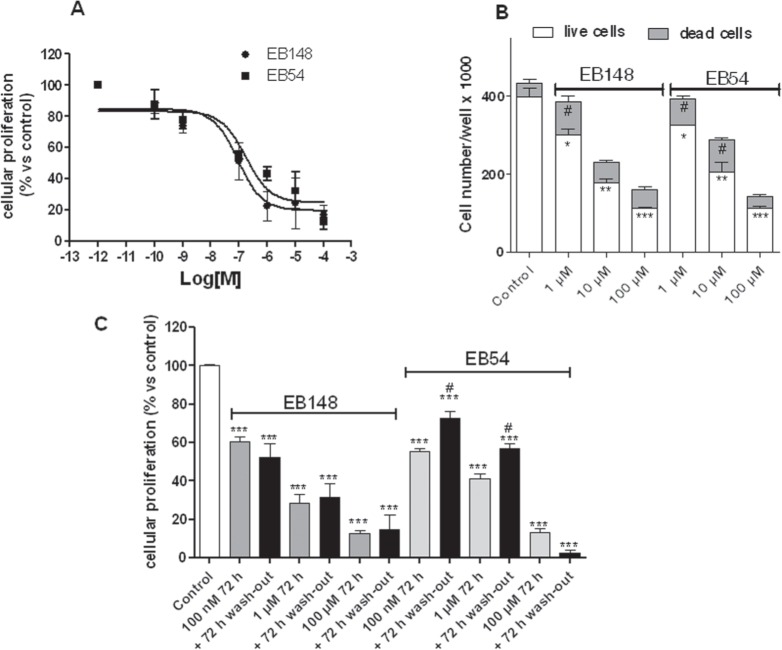
Effects of the MDM2 inhibitors on U87MG cell proliferation/viability **A**. U87MG cells were treated in complete medium with the indicated concentrations of EB148 or EB54 for 72 h. At the end of treatment, cell proliferation was measured using the MTS assay. The data are expressed as a percentage versus untreated cells (control), which was set to 100%, and are the mean values ± SEM of three independent experiments, each performed in duplicate. Curves were generated using a sigmoidal dose-response curve model (GraphPad Prism 5 software) from which the IC_50_ values were derived. **B**. U87MG cells were treated as in A; live and dead cells were then estimated using the trypan blue exclusion test. The data are expressed as number of living or dead cells per well and are the mean values ± SEM of two independent experiments, each performed in triplicate. * p<0.05, ** p<0.01, *** p<0.001 vs. control live cells; #p<0.05 vs. control dead cells. **C**. U87MG cells were treated with the indicated concentrations of EB148 or EB54 for 72 h, and then, the medium was replaced with drug-free fresh medium for additional 72 h. At the end of the treatment period, cell viability was measured using the MTS assay. The data are expressed as a percentage versus untreated cells (control), which was set to 100% and are the mean values ± SEM of three independent experiments, each performed in duplicate. The significance of the differences was determined with a one-way ANOVA with a Bonferroni post-test: *** p<0.001 vs. control; #p<0.05 vs. cells treated for 72 h.

The effects of the compounds on cell viability and proliferation were then examined. Cell treatment with EB54 or EB148 for 72 h induced a concentration-dependent reduction in cell proliferation, yielding IC_50_ values of 177 ± 15 nM and 91.6 ± 8.9 nM, respectively (Figure 11A). Moreover, both compounds reduced the total number of living cells, showing a maximal effect at 100 μM concentration (Figure 11B).

To assess whether U87MG cells could resume growing after inhibitor removal, culture medium was replaced with fresh medium not containing drugs and cell proliferation was monitored 72 h after cell wash-out. After EB148 cell treatment and subsequent removal, the inhibition of cell proliferation was maintained, thus suggesting the inability of the cells to restart proliferation. In contrast, a significant recovery in proliferative activity was detected when cells were pre-treated with the reversible compound EB54 (Figure 11C). These results suggest that the drug-target residence time plays a crucial role in the control of cell proliferation over-time.

To determine toxicity in non-tumour cells, the effects of EB148 and EB54 were examined on normal mesenchymal Stem Cells (MSCs) isolated from human bone marrow. Of note, the MSC viability of non-cancer cells was significantly decreased after 72 h of incubation with EB54 at 10 μM (Suppl. Figure 1). However, the percentages of cell proliferation reduction were significantly lower than those observed in U87MG cells, suggesting that the anti-proliferative effect elicited by the reversible dual ligand EB54 was directed preferentially towards tumour cells. In contrast, EB148 did not affect MSC proliferation even at the highest concentration tested, suggesting a lack of cell toxicity.

**Figure 12:**
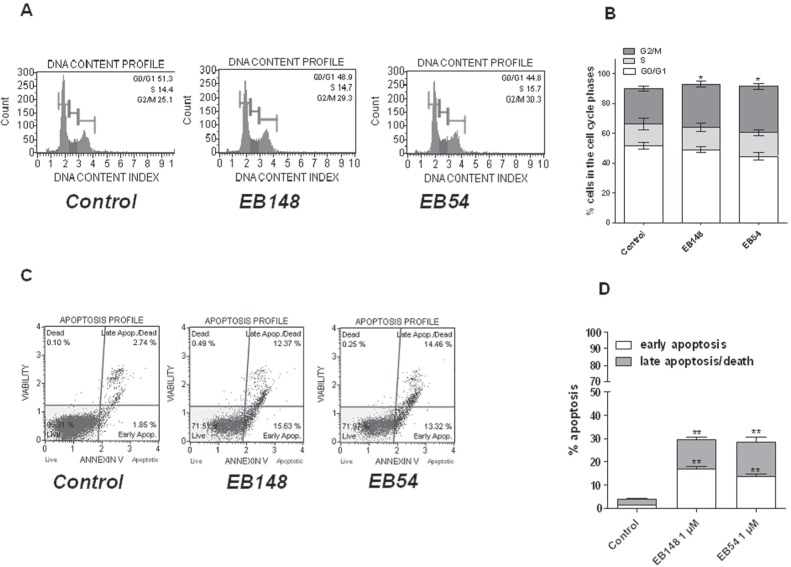
Effects of the MDM2 inhibitor on U87MG apoptosis and cell cycle **A**., **B**. U87MG cells were treated for 24 h with DMSO (control), or 1 μM EB148 or 1 μΜ EB54. At the end of the treatment period, the cell cycle was analysed as described in the Methods section. Representative cell cycle histograms of untreated and treated cells are shown in A. The data are expressed as the percentage of cells in the different phases (G0/G1, G2 or S) versus the total cell number and are the mean values ± SEM of three different experiments. The significance of the differences was determined with a one-way ANOVA with Bonferroni post-test: * p<0.05 vs. control in the respective cellular phase. **C**., **D**.) U87MG cells were treated as in A. At the end of the treatment period, the cells were collected, and the level of phosphatidylserine externalisation was evaluated using the Annexin V-staining protocol, as described in the Methods section. **D**. The data are expressed as the percentage of apoptotic cells (the data for the early-stage apoptotic cells shown in white, and the data for the late-stage apoptotic/necrotic cells shown in grey) versus the total number of cells. Data represent the mean ± SEM of three different experiments. The significance of the differences was determined with a one-way ANOVA with Bonferroni post-test: ** p<0.01 vs. control.

Then, the effect of EB148 on the regulation of cell cycle and on the apoptotic process was investigated. P53 activation in proliferating cells often caused cell cycle arrest at the G1 or G2 phases, an event that commonly involves the cyclin-dependent kinase inhibitor p21. According to these outcomes, a cell cycle cytofluorimetric assay revealed that both compounds similarly induced a significant increase in G0/G1 phase, suggesting a block of the cell cycle (Figure 12A and 12B).

In addition, the treatment of U87MG cells with EB148, as well as with the reversible EB54, induced a significant phoshpatidilserine externalization in the absence and in the presence of 7-amino-actinomysin binding to DNA, thus demonstrating that both compounds induced early and late apoptosis of the cells (Figure 12C and 12D).

## DISCUSSION

Compound EB148, designed as a dual molecule targeting both MDM2 and TSPO proteins, was demonstrated in this study to activate the ERK intracellular signalling pathway over time, driving GBM cells towards an unavoidable fate of death. These effects were also maintained after ligand depletion, thus suggesting that increasing the drug-residence time to its targets may represent a proper way to improve GBM treatment outcome.

GBM treatment is an undisputed neurosurgical challenge. The major limitations of the current anti-proliferative therapies for GBM are related to the onset of drug resistance phenomena [5]. Results from both basic and clinical research have suggested that the development of irreversible agents [12, 19] and multi-target compounds [9] may lead to the discovery of novel therapeutic strategies for GBM and other types of solid tumours. In terms of current multi-therapy strategies, dual PI3K/mTOR inhibitors are emerging in GBM. Several inhibitors targeting these two kinases have been developed; for example, XL765 has been demonstrated to reduce cell viability *in vitro* and is possibly effective when combined with TMZ therapy [33]. Recently, we identified new reversible compounds dual-targeting MDM2 and TSPO, two proteins that are both up-regulated in GBM so contributing to cancer cell resistance to physiological apoptosis [25]. These compounds have showed high and prolonged anti-proliferative activity in GBM cells, with significantly higher effects than those elicited by the single target reference standards, thus confirming that dual inhibitors might have improved results compared to monotherapy.

Furthermore, it is also true that targeting one or more signalling pathways with reversible molecules may be not enough to sustain the therapeutic effects over time, and actually may favour the activation of alternative signalling pathways and the onset of drug resistance phenomena. Recently, there has been a resurgence of interest towards irreversible inhibitors, and this topic has been excellently reviewed in several publications from a risk-benefit perspective [34, 35] and in terms of the current irreversible inhibitors that are in preclinical or clinical development [36]. Several tyrosine kinase inhibitors with irreversible activity have been developed, and some of these are now in phase I-III trials for the treatment of different solid tumours, [37-40] including GBM. The irreversible inhibitors include Canertinib (CI1033; Pfizer/Warner-Lambert), Pelitinib (EKB-569; Wyest-Ayerst) and Dacomitinib [41].

Based on this evidence, we wondered whether a molecule that modulated two distinct intracellular targets (namely MDM2 and TSPO) with a long-lasting mechanism of action, might have greater and longer life span of anti-proliferative activity in GBM cells.

In the design of the new irreversible dual target compound, the basic structure of the recently developed 2-phenylindol-3ylglyoxyldipeptide derivative EB54, [25] was examined to determine the best synthetically feasible position for the introduction of a moiety conferring long-lasting properties. The 5-position of the indole ring seemed suitable for a chemo-reactive group. Among possible chemo-reactive moieties, isothiocyanate has proven extremely versatile as an electrophilic moiety for long-lasting ligands. It can easily be synthesized from a primary amino group; furthermore, its high reactivity towards amino and sulfhydryl groups, along with its low reactivity towards water and other hydroxyl functions, accounts for its successful applications in receptor studies [26, 42]. Actually, we recently employed this moiety to develop selective irreversible TSPO ligands as useful tools to study the role of this protein in human GBM cells [26, 43]. Thus, the derivative EB148 was synthesized and biologically evaluated.

Compound EB148 was able to cause GBM cell death by arresting the cell cycle and inducing an apoptotic pathway of cell death. The effects elicited by EB148 appeared to be greater and more long-lasting than those of the reversible analogue. Furthermore, the apoptotic effects appeared to be irreversible so that the cells were not able to regain proliferative activity after drug wash-out.

The biological characterization of EB148 started with the evaluation of its ability to bind TSPO and to induce Δψm collapse in mitochondria isolated from GBM cells. The compound displayed a nanomolar range affinity for TSPO, with a long-lasting binding profile, as demonstrated by kinetic competition experiments. Through TSPO activation, EB148 induced permeability transition pore opening in GBM cells without any steroidogenic activity and, differently to that which occurred with the reversible analogue EB54, this effect was maintained over time, even after cell wash-out. Therefore, we can conclude that the long-time activation of TSPO caused an irreversible mitochondrial collapse.

Then, the ability of the same compound to dissociate the MDM2-p53 complex was investigated by an *in vitro* ELISA-based assay [25]. To evaluate the covalent mechanism of action, kinetic dissociation studies of p53-MDM2 complex inhibition were performed both in cell lysates and in whole cells. EB148 inhibited MDM2-p53 association with a nanomolar potency, a value comparable to that detected with the reversible analogue EB54. As a major difference, the long-lasting compound EB148 induced prolonged inhibition of the MDM2-p53 complex that was maintained even after cell wash-out, thus demonstrating its covalent binding to MDM2 protein. The sustained inhibition of MDM2-p53 complex formation may account for the different kinetic pattern in the regulation of p53 gene targets induced by the reversible versus the long-lasting compound and may explain the differences in the long-term pro-apoptotic effects elicited by EB148. To dissect the molecular mechanism involved in the long-term effects of EB148, the modulation of ERKs was investigated. The GBM biological characteristics are exemplified by prominent proliferation, active invasiveness and rich angiogenesis. These features are mainly due to highly deregulated signalling pathways in the tumour, in particular the Ras/MEK/MAPK pathways [44].

MAPK signalling plays a crucial role in almost all cell functions and therefore requires exquisite control of its spatiotemporal activity [45]. Depending on the duration, the magnitude and its subcellular localization, ERK activation controls various and opposite cell responses, such as proliferation, migration, differentiation and death.

An interesting question is how such an apparently straightforward and simple cascade can generate a wide array of biological responses depending on the cellular context. One way to achieve diversity in signalling outcomes is to control the signal duration by acting on the dose and on the drug-target residence time. Indeed, limiting exposure to ligands could be another mechanism to control signalling duration and switch-like cellular responses [46, 47].

The proliferative effects of ERKs in response to mitogenic signals are commonly associated with a transient activation of these kinases. In contrast, a sustained ERK activation induces a progressive accumulation of death-promoting factors, up to a level that induces cell death. Prolonged ERK activation has been shown to promote death of human cancer cell lines of different origins and to be the primary mechanism of action of different chemotherapy agents [48-53]. Understanding the critical pathological roles of this signalling molecule and its regulatory mechanisms in the progression of glioma is important for the development of effective molecular-targeted therapies against GBM. Furthermore, the development of ERK signalling modulators may likely represent a smart strategy to selectively target cancer cells with deregulated ERK activity but not normal cells in which ERK activation is transient.

In several tumour cell lines, a strict interplay between ERK signalling and the MDM2-p53 pathway has been demonstrated. ERK, via a phophorylation process, promotes p53 stability and activity and inhibits p53 interaction with MDM2. On the other hand, p53 may act as an upstream regulator of ERK activation for the drug-mediated induction of apoptosis in different tumour cells [46, 48, 51]. Notwithstanding that the role of the p53-MDM2 axis in the progression of GBM has been well explained, the interplay between p53-MDM2 and the ERK pathway has not yet been deeply investigated.

In this study, EB148 was demonstrated to induce a sustained and p53-dependent stimulation of ERK1/2 phosphorylation, whereas no significant activation was detected after treatment of cells with the reversible analogue, EB54. Because of prolonged ERK activation, EB148 favoured the association of MDM2 with ERK. Because it has been demonstrated that ERK mediated MDM2 phosphorylation reduced the ubiquitin ligase activity of MDM2 and in turn potentiated p53 pro-apoptotic effects, we can speculate that EB148, favouring MDM2-ERK association, contributed to sustain p53 activation over time. In this scenario, the long-lasting inhibition of MDM2 may activate a feed-back mechanism through which the p53-MDM2-ERK axis feeds itself in the control of cell fate triggering an irreversible path of cell death.

Overall, our data demonstrated that in GBM cells 1) EB148 acts as a dual and irreversible-like ligand targeting both MDM2 and TSPO proteins, 2) the long-lasting activation of the two targets induced irreversible apoptosis, and 3) the ERK pathway is likely one of the molecular mechanism involved in the p53-mediated effects.

## MATERIAL AND METHODS

### Reagents and cells

The human glioblastoma multiforme U87MG cells were obtained from the National Institute for Cancer Research of Genova (Italy) and monitored for DNA profiling. Propidium iodide (PI) and the fluorescent dye, 5,50,6,60-tetrachloro-1,10,3,30-tetraethylbenzimidazolcarbocyanine iodide (JC-1) were obtained from Molecular Probes, Invitrogen, Milan, Italy. The 3-(4,5-dimethylthiazol-2-yl)-5-(3-carboxymethoxyphenyl)-2-(4-sulfophenyl)-2H-tetrazolium (MTS) assay kit was from Promega Italia, Milan, Italy. The RNeasyH Mini Kit was from Qiagen, Milan, Italy and the ProtoScriptH cDNA Synthesis Kit was obtained from Biolabs, Euroclone, Milan, Italy.

### Chemical synthesis

General directions. A Reichert Kofler hot-stage apparatus was used to determine the melting points, that are uncorrected. For routine nuclear magnetic resonance spectra, compounds were dissolved in DMSO-d_6_ and a Bruker operating at 400 MHz was utilized. Analytical TLC was carried out on Merck 0.2 mm precoated silica gel aluminum sheets (60 F-254).

*N*-Boc-L-leucine-L-phenylalanine ethyl ester 2, [25] L-leucine-L-phenylalanine ethyl ester 3, [25] 5-nitro-2-phenylindole 5, [26] and 5-nitro-(2-phenylindol-3-yl)glyoxyl chloride 6 [26] were prepared in accordance with our previous works.

*(5-Nitro-2-phenylindol-3-yl)glyoxyl-L-leucine-L-phenylalanine ethyl ester* 7. Oxalyl chloride (0.43 ml, 5.0 mmol) was added dropwise, at 0°C, to a well-stirred mixture of the 5-nitro-2-phenylindole 5 (2.5 mmol) in freshly distilled diethyl ether (10 ml). The mixture was maintained at room temperature for 12 h. The precipitate was collected by vacuum filtration to give the acyl chloride 6 that was directly used in the subsequent reaction. A solution of compound 3 (0.360 g, 1.09 mmol) in 5 ml of dry toluene was added dropwise to a stirred suspension, cooled at 0°C, of the 5-nitro-2-phenylindol-3-ylglyoxylyl chloride 6 (1.23 mmol) in 15 ml of the same solvent, followed by the addition of a solution of triethylamine (0.18 ml, 1.31 mmol) in 1.5 ml of dry toluene. The reaction mixture was left under stirring overnight at room temperature (TLC analysis) and then filtered. The collected precipitate was washed with a 5% NaHCO_3_ aqueous solution and collected again to give a first portion of crude product. The toluene was removed under reduced pressure and the residue dissolved with CH_2_Cl_2_. The organic solution was washed with diluted HCl, a 5% NaHCO_3_ aqueous solution and water, dried (MgSO_4_), and evaporated to dryness to yield an additional amount of crude product. Compound 7 was finally purified by flash-chromatography (AcOEt: hexane = 4:6 as eluent). Yield 74%; mp = 110-112°C. ^1^H NMR (400 MHz, DMSO-d_6_) δ (ppm): 0.82 (d, *J =* 6.36 Hz, 6H); 1.06 (t, *J =* 7.2 Hz, 3H); 1.22-1.29 (m, 1H); 1.36-1.49 (m, 2H); 2.96-2.99 (m, 2H); 3.97 (q, *J =* 7.0 Hz, 2H); 4.15-4.22 (m, 1H); 4.43-4.47 (m, 1H,); 7.22-7.28 (m, 5H); 7.45-7.47 (m, 3H); 7.62-7.65 (m, 2H); 7.68 (d, *J* = 8.8 Hz, 1H); 8.18 (dd, *J* = 9.2 Hz, *J* = 2.4 Hz, 1H); 8.45 (d, *J* = 7.2 Hz, 1H, exch. with D_2_O); 8.74 (d, *J* = 8.4 Hz, 1H, exch. with D_2_O); 8.92 (d, *J =* 2.4 Hz, 1H); 13.00 (bs, 1H, exch. with D_2_O).

*(5-Amino-2-phenylindol-3-yl)glyoxyl-L-leucine-L-phenylalanine ethyl ester* 8. A mixture of (5-nitro-2-phenylindol-3-yl)glyoxyl-L-leucine-L-phenylalanine ethylester 7 (0.200 g, 0.33 mmol) and 10% Pd/C (0.050 g) in 150 ml of absolute ethanol was hydrogenated at room temperature. Once hydrogen absorption ceased (3-4 h), the catalyst was filtered off and the solvent was evaporated. Compound 8 was finally purified by flash-chromatography (AcOEt: hexane = 6:4 as eluent). Yield 71%; mp = 98-100°C; ^1^H NMR (400 MHz, DMSO-d_6_) δ (ppm): 0.79 (d, *J =* 6.4 Hz, 6H); 1.06 (t, *J =* 7.2 Hz, 3H); 1.11-1.15 (m, 1H); 1.32-1.44 (m, 2H); 2.90-3.02 (m, 2H); 3.97-4.05 (m, 3H); 4.43-4.47 (m, 1H); 4.87 (bs, 2H, exch with D_2_O); 6.61 (dd, *J =* 2.4 Hz, *J =* 8.8 Hz, 1H); 7.16 (d, *J =* 8.8 Hz, 1H); 7.20-7.23 (m, 3H); 7.26-7.29 (m, 2H); 7.34-7.38 (m, 4H); 7.49-7.51 (m, 2H); 8.33 (d, *J =* 7.2 Hz, 1H, exch with D_2_O); 8.41 (d, *J =* 8.8 Hz, 1H, exch with D_2_O); 11.92 (bs, 1H, exch with D_2_O).

*5-[N-(5-Isothiocyanato-2-phenylindol-3-yl)glyoxyl-L-leucine-L-phenylalanine ethyl ester* EB148. A stirred solution of 8 (0.200 g, 0.35 mmol) in 20 ml of 6% NaHCO_3_ was added to 20 ml of CH_2_Cl_2_. After 20 min, thiophosgene (0.03 ml, 0.35 mmol) was added dropwise at 0°C. The reaction mixture was stirred overnight. The aqueous phase was then extracted with CH_2_Cl_2_ and the resulting organic layer was dried over MgSO_4_. The product was finally purified by flash chromatography (Hexane: AcOEt = 6:4 as eluent). Yield 62%; mp = 93-95°C. ^1^H NMR (400 MHz, DMSO-d_6_) δ (ppm): 0.84 (d, *J* = 6.4 Hz, 6H); 1.07 (t, *J* = 7.2 Hz, 3H); 1.24-1.33 (m, 1H); 1.36-1.51 (m, 2H); 2.97-3.01 (m, 2H); 3.99 (q, *J* = 7.2 Hz, 2H); 4.17-4.23 (m, 1H); 4.48-4.53 (m, 1H); 7.21-7.24 (m, 5H); 7.34 (dd, *J* = 8.4 Hz, *J* = 2.0 Hz, 1H); 7.43-7.46 (m, 3H); 7.54 (d, *J* = 8.4 Hz, 1H); 7.60- 7.62 (m, 2H); 7.95 (d, *J* = 2.0 Hz, 1H); 8.43 (d, *J* = 7.2 Hz, 1H, exch with D_2_O); 8.68 (d, *J* = 8.4 Hz, 1H, exch with D_2_O); 12.67 (bs, 1H, exch with D_2_O). ^13^C NMR (100 MHz, DMSO-d_6_) δ (ppm): 14.32; 22.60; 23.17; 24.43; 37.14; 41.40; 51.07; 54.03; 60.90; 109.46; 113.91; 118.49; 121.78; 124.63; 126.98; 128.10; 128.67; 129.53; 129.94; 130.09; 131.33; 135.07; 137.42; 149.09; 165.94; 171.65; 171.69; 186.77.

### Stability test

To test the stability of EB148 in aqueous solution, the compound was dissolved in DMSO and diluted with PBS to the final 1 μM concentration (the amount of DMSO in the final solution was 1%). The solution was maintained in a closed vial and analysed by HPLC at specific times (4, 8, 24, 48 and 72 h) using the following conditions: column Shim-pack VP-ODS 4.6 μm (250 mm × 4.6 mm), isocratic elution with water, flow rate of 1 ml/min, room temperature. The percentage of decomposition was calculated on the base of peak area of the product.

### Cell cultures

U87MG cells were cultured in RPMI medium supplemented with 10% FBS, 2 mM-glutamine, 100 U/ml penicillin, 100 mg/ml streptomycin and 1% non-essential amino acids at 37°C in 5% CO_2_. U87MG cells were plated at 5×10^3^ cells/cm^2^. After 24 h, culture medium was replaced with fresh medium containing newly synthesized compounds solubilized in DMSO for the indicated incubation times. DMSO was added to control cells (0.5 % v/v). Human MSCs were cultured in normal growth medium (MSCGM, Lonza), plated (5×10^3^ cells/cm^2^) in 75-cm^2^ flasks and incubated at 37°C in 5% CO_2_ and 95% air. The medium was changed to remove non adherent cells every 3 to 4 days, and the cells were used at passages 0 to 3.

### TSPO binding assays

Equilibrium radioligand binding assays were performed essentially as previously described [54]. Briefly, aliquots of U87MG cell membranes (20 μg of proteins) were incubated with [^3^H]PK 11195 (1.5 nM) in the presence (non-specific binding) or in the absence (total binding) of unlabelled PK 11195 (1 μM), in the final volume of 500 μl of assay buffer for 120 min at 0°C. Samples were rapidly filtered under vacuum through GF/C glass fiber filters. Radioactivity was measured by liquid scintillation counter (TopCount; PerkinElmer Life and Analytical Sciences; 65 % counting efficiency). In U87MG cell membranes, [^3^H]PK11195 maximum number of binding sites (Bmax) and affinity (Kd) were determined in previous experiments by Scatchard analysis of saturation binding data [43].

### TSPO association and dissociation kinetics

Association kinetics for [^3^H]PK11195 were determined incubating U87MG cell membranes (20 μg protein) with 1.5 nM radioligand at various time points up to a total of 3 h. Dissociation kinetics were determined by pre-equilibrating membranes and [^3^H]PK11195 for 120 min and the adding a saturating concentration of cold PK11195 (1 μM).

Association and dissociation rate constants for [^3^H] PK11195 binding were determined by global analysis of the association and dissociation binding curves according to mono-exponential association and decay curves. From this analysis an estimate of the dissociation (koff) and observed association (kobs) rates for ligand binding was generated; the association rate constant (kon) was determined according to the following relationship *kobs* = [*A*] *kon* + *koff;* ligand-receptor half-life was calculated as 0.693/koff.

For competition kinetics, the [^3^H] PK11195 curves in the presence and absence of EB54 or EB148, were fitted using the equations in Dowling and Charlton [55].

### Mitochondrial membrane potential (Δψm) dissipation analysis

The Δψm dissipation was assessed using the fluorescent dye 5,59,6,69-tetrachloro1,19,3,39-tetraethylbenzimidazolylcarbocyanine iodide (JC-1) [25]. Mitochondria were isolated from U87MG cells using the Mitochondria Isolation Kit (Sigma Aldrich, Milan, Italy) following manufacturer's instructions.

Isolated mitochondria were treated for 20 min with EB54 or EB148 (1 μM). In wash-out experiments, mitochondria were then centrifuged at 11.000 × g for 5 min, washed with fresh saline and suspended in JC-1 buffer not containing the drugs. At the end of treatments, 5 μg of control and treated mitochondria were added to the JC-1 dye. The fluorescence (relative fluorescence units, RFU) of the sample was read in a spectrofluorimeter using a time-drive method (one acquisition each 10 sec, for 10 min), where the excitation wavelength was set to 490 nm and emission wavelength at 590 nm. JC-1 exhibits potential-dependent accumulation in mitochondria, where higher concentrations start to form J-aggregates indicated by a fluorescence emission shift from green (529 nm, monomer emission maximum) to orange-red (590 nm, aggregate emission maximum). Consequently, mitochondrial depolarization is indicated by a decrease in the orange-red fluorescence intensity.

### Dissociation studies of native MDM2/p53 complex

To test the ability of new compound to dissociate the native MDM2/p53 complex, a quantitative sandwich immune-enzymatic assay, on crude cell lysates obtained from U87MG cells was applied [25, 29]. Cells were washed twice in ice-cold phosphate-buffered saline, collected by centrifugation, and suspended in lysis buffer (20 mM Tris HCl, 137 mM NaCl, 10% glycerol, 1% NONIDET40, 2 mM EDTA, pH 8) containing 1% of the Protease inhibitor Cocktail (Sigma Aldrich, Milan, Italy). 96-wells were pre-coated with a mouse full-length anti-MDM2 antibody (sc-965, Santa Cruz Biotechnology, 1:50 in 0.05% Poly-L-Ornithine) overnight at room temperature. Cell lysates (20 μg in a final volume of 100 μl) were pre-incubated with DMSO (control), or different compound concentration for 10 min at room temperature, and then transferred to the pre-coated wells for 60 min. After three quick washes with PBS/Tween 0.05% to remove unbound MDM2, each well was incubated for 15 min with 1% BSA, to block nonspecific sites, and then for 1.5 h at room temperature with a rabbit primary anti-p53 antibody (sc-6243, Santa Cruz Biotechnology, 1:250). Then, wells were washed and incubated for 1 h with an anti-rabbit HRP-conjugate antibody, and washed again. The TMB substrate kit (Thermo Fisher Scientific) allowed a colorimetric quantification of the MDM2/p53 complex. Blanks were obtained processing cell lysates in the absence of the primary anti-p53 antibody. Absorbance's values at 450 nm were measured, background subtracted and sigmoid dose-response curves were generated using Graph Pad Prism 4 software, from which IC_50_ values of MDM2/p53 complex were derived.

### EB148 binding to MDM2: association and dissociation kinetics

To investigate the association kinetics of the compounds to human MDM2, 20 μg of U87MG cell lysates, suspended in lysis buffer, were incubated for different times (0-120 min) with EB148 or EB54. Following incubation, cell lysates were captured on wells pre-coated with MDM2 antibody for an additional 60 min. After extensive washes, levels of the MDM2/p53 complex were quantified as described above.

In dissociation studies, U87MG cell lysates, suspended in lysis buffer, were incubated for 90 min with EB148 or EB54 in order to reach association plateau. Dissociation was initiated by the addition of 20 volumes of cold buffer, and lysates were further incubated at 4°C for the indicated times (0-180 min). Following incubation, 200 μl of the mixture were captured on wells pre-coated with MDM2 antibody for an additional 60 min. After extensive washes, levels of the MDM2/p53 complex were quantified as described above.

### Quantitation of p53/MDM2 and ERK/MDM2 complexes in U87MG cells

U87MG cells were treated with DMSO (control), EB148 or EB54 for different times. When indicated, after 8 h of incubation, cells were washed twice with fresh saline and cultured in drug-free medium for an additional 24 h. At the end of the treatment period, the cells were collected, and suspended in lysis buffer containing 1% of the Protease inhibitor Cocktail. Levels of p53/MDM2 or ERK/MDM2 complexes were quantified using the ELISA test method described above in wells pre-coated with an anti-MDM2 antibody. Cell lysates (20 μg in a final volume of 100 μl) were transferred to the pre-coated wells for 60 min; each well was then incubated for 1.5 h at room temperature with p53 or ERK (sc-7383 SantaCruz Biotechnology, 1:500) primary antibodies.

### Relative mRNA quantification of p53 target genes

The assessment of the levels of p53 mRNA gene targets was evaluated by real-time reverse transcription polymerase chain reaction (real-time RT-PCR). In brief, total RNA was extracted from control cells as well as from cells treated with EB148 or EB54 (1 μM) for different times (8 h, 16 h or 24 h), using RneasyH Mini Kit (Qiagen, Hilden, Germany), according to manufacturer's instructions. cDNA synthesis was performed with 500 ng of RNA using using i-Script cDNA synthesis kit (BioRad, Hercules, USA). RT-PCR reactions consisted of 25 μl FluocycleH II SYBRH (Euroclone, Milano, Italy), 1.5 μl of both 10 μM forward and reverse primers (listed in [56]), 3 μl cDNA, and 19 μL of H_2_O. For each sample, mRNA levels of p53 gene target were normalized against β-actin mRNA levels, and relative expression was calculated by using Ct value. PCR specificity was verified by both the melting curve analysis and gel electrophoresis.

### Western blotting analysis

U87MG were treated with DMSO (control), EB148 or EB54 for 16 h. When indicated, after 16 h of incubation, cells were washed twice with fresh saline, and incubated with fresh medium not containing the drugs for an additional 24 h. At the end of the treatment period, the cells were collected and then lysed for 60 min at 4°C using 200 μl of RIPA buffer (9.1 mM NaH_2_PO_4_, 1.7 mM Na_2_HPO_4_, 150 mM NaCl, pH 7.4, 0.5% sodium deoxycholate, 1% Nonidet P-40, 0.1% SDS, and a protease-inhibitor cocktail). Equal amounts of the cell extracts (40 μg of protein) were diluted in Laemmli sample solution, resolved using SDS-PAGE (8.5%), transferred to PVDF membranes and probed overnight at 4°C using an anti-p53 antibody (FL-393, Santa Cruz Biotechnology; 1:500). Glyceraldehyde-3-phosphate dehydrogenase (GAPDH) was the loading control (G9545, Sigma Aldrich, Milan, Italy, 1:5000). The primary antibodies were detected using the appropriate peroxidase-conjugated secondary antibodies, which were then detected using a chemioluminescent substrate (ECL, Perkin Elmer). Densitometric analysis of the immunoreactive bands was performed using Image J Software.

### Cell viability and proliferation assay

The effects of compound treatment on U87MG cell viability and proliferation were evaluated by Trypan blue exclusion assays and MTS assay, respectively. U87MG cells were treated with EB148 or EB54 for 72 h. Following treatment, harvested cells were mixed with an equal volume of 0.4% trypan blue dye. For quantization of cell viability, blue and bright cells were counted, and viability was calculated as the number of living (bright) and dead (blue) cells in each well. To establish the effect of MDM2 inhibitors on cell proliferation, cells were treated for 72 h with different concentrations of each compound (0.1 nM–100 μM) for 72 h, and then MTS assay was performed according to the manufacturer's instructions.

The absorbance of formazan at 490 nm was measured in a colorimetric assay with an automated plate reader (Victor Wallac 2, Perkin Elmer). For wash-out experiments, U87MG cells were treated with EB148 or EB54 (100 nM-10 μM) for 72 h. Following incubation, medium-containing drugs was replaced by fresh medium, and cells were allowed to growth for the indicated days. At the end of treatments, cell proliferation was measured by MTS assay. The results were calculated by subtracting the mean background from the values obtained from each evaluation and were expressed as the percentage of the control (untreated cells). Sigmoid dose-response curve was generated, from which the IC_50_ values were derived.

To evaluate compound toxicity, MSCs were treated for 72 h with different concentrations of EB148 or EB54. At the end of treatments, cell proliferation was measured by MTS assay.

### Cell cycle analysis

The measurement of the percentage of cells in the different phases of cell cycle was performed using the MuseTM Cell Analyzer (Merck KGaA, Darmstadt, Germany). Briefly, U87MG cells were treated for 24 h with DMSO, reversible or irreversible compound (1 μM). Adherent cells were collected and suspended in the fluorescent reagent (MuseTM Cell Cycle reagent). After incubation for 30 min at room temperature in the dark, the measurements of the percentage of cells in the different phases was acquired [25].

### Annexin V and 7-AAD staining

Dual staining with Annexin V coniugated to fluorescein-isothiocyanate (FITC) and 7-amino-actinomysin (7-AAD) was performed using the commercially available kit (Muse Annexin V and Dead Cell Kit; Merck KGaA, Darmstadt, Germany).

U87MG cells were treated with DMSO (control), EB54 or EB148 (1 μM) for 24 h. Both floating and adherent cells were collected, and incubated for 10 min at room temperature with the fluorescent reagent. After incubation, the percentages of living, apoptotic and dead cells were acquired and analyzed by MuseTM Cell Analyzer in accordance to the manufacture's guidelines [25].

### Statistical analyses

The nonlinear multipurpose curve-fitting program Graph-Pad Prism (GraphPad Software Inc., San Diego, CA) was used for data analysis and graphic presentations. All data are presented as the mean ± SEM. Statistical analysis was performed by one-way analysis of variance (ANOVA) with Bonferroni's corrected t-test for post-hoc pair-wise comparisons. P < 0.05 was considered statistically significant.

## SUPPLEMENTARY MATERIAL FIGURE


