# Supplementary material for: Long lasting MDM2/Translocator protein modulator: a new strategy for irreversible apoptosis of human glioblastoma cells
Source: Oncotarget. 2016 Jan 9;7(7):7866–84. doi: 10.18632/oncotarget.6872 (PMC4884960; doi:10.18632/oncotarget.6872)
Supplement: Supplementary file 1 [file oncotarget-07-7866-s001.pdf]

# Long lasting MDM2/Translocator protein modulator: a new strategy for irreversible apoptosis of human glioblastoma cells

**Supplementary Material**

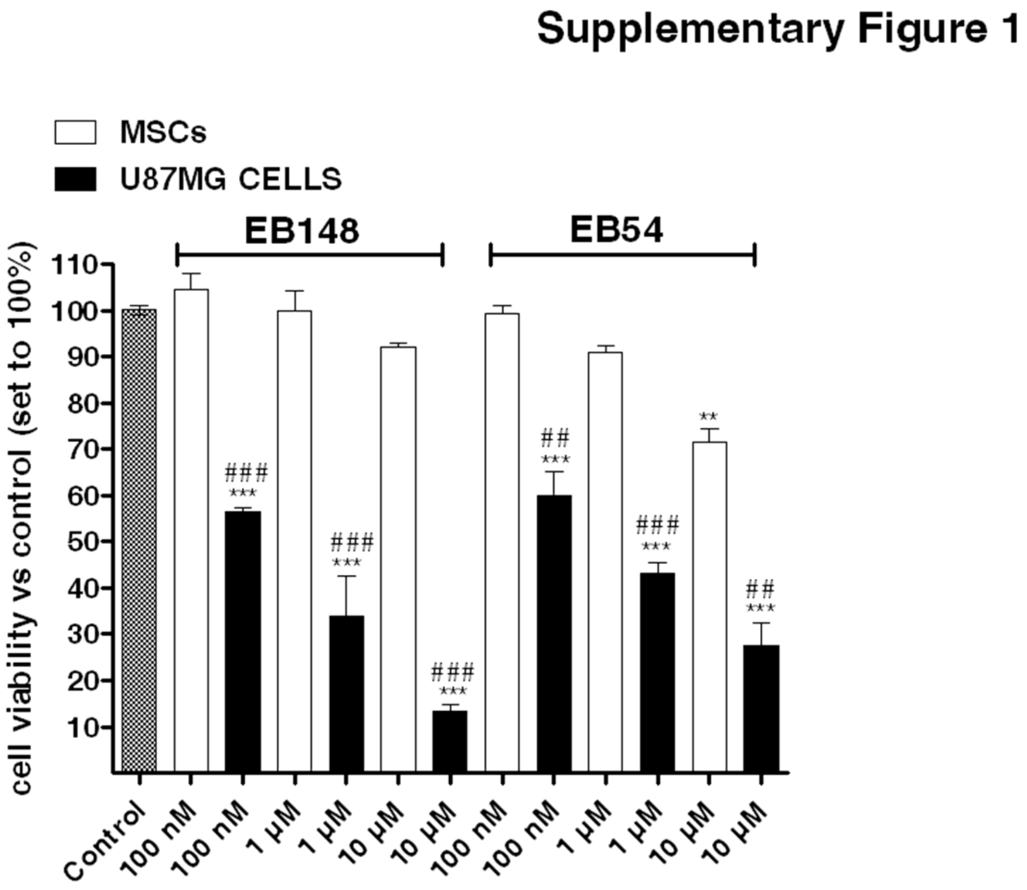


**Supplementary Figure 1.** MSCs or U87MG cells were incubated with the indicated concentration of EB148 or EB54 for 72 h. At the end of treatment, cell proliferation was measured using MTS assay. The data are expressed as a percentage with respect to that of untreated cells (control), which was set to 100%, and are the mean values ± SEM of three independent experiments, each performed in duplicate. The significance of the differences was determined with a one-way ANOVA with Bonferroni post-test: *** p<0.001 vs control; ## p<0.005, ### p<0.001 vs. percentages in MSCs.
